# Triterpene and Steroids from *Ludwigia abyssinica* A. Rich (Onagraceae) Displayed Antimicrobial Activities and Synergistic Effects with Conventional Antibiotics

**DOI:** 10.1155/2023/2975909

**Published:** 2023-04-10

**Authors:** Arlette Meli Sonkoue, Irene Chinda Kengne, Stephen Tamekou Lacmata, Claudia Darille Jouogo Ngnokam, Mahamat Djamalladine Djamalladine, Laurence Voutquenne-Nazabadioko, David Ngnokam, Jean-de-Dieu Tamokou

**Affiliations:** ^1^Research Unit of Applied and Environmental Chemistry, Department of Chemistry, Faculty of Science, University of Dschang, P.O. Box 67, Dschang, Cameroon; ^2^Research Unit of Microbiology and Antimicrobial Substances, Department of Biochemistry, Faculty of Science, University of Dschang, P.O. Box 67, Dschang, Cameroon; ^3^Groupe Isolement et Structure, Institut de Chimie Moléculaire de Reims (ICMR), CNRS UMR 7312, Bat. 18 B.P. 1039, 51687 Reims Cedex 2, Reims, France

## Abstract

Difficulties encountered in treating drug-resistant pathogens have created a need for new therapies. Synergistic combinations of antibiotics are considered as ideal strategies in combating clinical and multidrug-resistant (MDR) infections. In this study, the antimicrobial activities of triterpenes and steroids from *Ludwigia abyssinica* A. Rich (Onagraceae) and their combined effects with antibiotics were assessed. The associations between plant constituents and antibiotics were evaluated by determining their fractional inhibitory concentrations (FICs). Sitost-5-en-3*β*-ol formiate (**1**), 5*α*,6*β*-dihydroxysitosterol (**2**), and maslinic acid (**3**) were isolated from the *L. abyssinica* ethyl acetate (EtOAc) extract. The EtOAc extract, compounds **1, 2,** and **3** (MIC = 16–128 *µ*g/mL) would be the best antibacterial and antifungal agents. The antimicrobial activities of amoxicillin were relatively weak against MDR *Escherichia coli* and *Shigella flexneri* and significant against *Staphylococcus aureus* ATCC 25923. However, when used in association with plant constituents, it displayed an interesting synergistic effect. Among plant components-antibiotic combinations, the EtOAc extract and compound **1** (steroid) showed a synergistic effect with amoxicillin/fluconazole against all the tested microorganisms whereas the association of compound **3** (triterpenoid) and amoxicillin/fluconazole displayed an additive effect against *Shigella flexneri* and *Escherichia coli* and a synergistic effect on *Staphylococcus aureus*, *Cryptococcus neoformans*, *Candida tropicalis*, and *Candida albicans* ATCC 10231. Overall, the results of the present study demonstrated antibacterial and antifungal activities of extracts and compounds isolated from *L. abyssinica.* The findings of the current study also showed that the potency of antibiotics was improved when screened in combination with *L. abyssinica* components, supporting the drug combination strategy to combat antimicrobial resistance.

## 1. Introduction

Regardless of the significant progress noted in medical sciences, infections are still the main cause of illnesses and deaths worldwide [1]. Due to the often inappropriate use of antibiotic drugs by the population, microbes have developed resistance strategies which have culminated to treatment failure and further increased the cost of treatment. The mode of action of antibiotic resistance, stemming from long duration of use, is the generation of multiple novel genes, with resistant attributes to one drug [2]. This mechanism of action in a bacterial cell has culminated in the evolution of bacteria with multiple resistances to drugs. Some diseases caused by MDR bacterial strains are without cure and are deadly as a result of the increased resistance rate in most of the therapeutic antibiotics [3].

Some methods have been developed to counter bacterial drug resistance. Generating new antibiotic substances is one common approach. Nonetheless, making new antibacterial substances can be challenging and new resistance tactics will arise following the large clinical use. A new way to counter the resistance is implementing combination therapy, where at least two antibacterial drugs are used to strengthen antibacterial actions against the resistant infectious microbes. Chemical substances obtained from plants are great sources for therapeutic combinations. The natural triterpenes and steroids are documented for their antibiotic attributes and activity. Proof of this is considered thoughtfully through their destructive effects on the microbial structure as well as functional interruption on cell membranes and cell walls [4]. An effective strategy to overwhelm antimicrobial drug resistance, chemical damage to cell, and prolong treatments with the antibiotics at hand is combinatorial therapy or synergistic interaction. Drug action effects in killing microbes such as inhibition, addition, or synergy have been observed following the combinations of triterpenes and antibiotics. For instance, a synergistic effect between oleanolic acid and ethambutol, rifampicin, or isoniazid targeting *Mycobacterium tuberculosis* have been reported in vitro [5]; while synergism has also been observed in many triterpenoids (betulinic acid, imberbic acid, rotundic acid, ulsolic acid, oleanic acid, beta-boswellic acid, and cycloastragenol)-antibiotic combinations targeting multiresistant *Staphylococcus aureus* variants [6, 7]. Due to increasing antimicrobial resistance, the search for alternative compounds with potential microbicidal effects has become top-priority. In the traditional practice of medicine, plants are commonly used as therapeutic sources to treat infections.

*Ludwigia* is a large genus within the Onagraceae family, which is widespread in both temperate regions such as Europe, tropical and subtropical regions like Africa, Australia, and the West Indies [8]. Many species belonging to this genus constitute plants used for medicines in the traditional sector of practice [9, 10]. Early reports on the *Ludwigia* genus highlighted the presence of bioactive secondary metabolites such as flavonoids [11, 12], cerebrosides, and triterpenoids [12, 13]. Many of them possess various pharmacological properties such as antidiarrheal [3], antitumoral and antibacterial [13, 14], antimicrobial, and antioxidant [15, 16] properties. Furthermore, recent studies have demonstrated that alcohol extracts of *L. abyssinica* leaves as well as those of *L. octovalvis* and *L. decurrens* do possess antioxidant, antimycotic, and antibacterial attributes [8, 17]. These studies revealed the antimicrobial potential of *L. abyssinica* but without any information on its chemical components as well as antimicrobial effects. As we seek for compounds with the microbe-interruptive biological activity from Cameroon medicinal plants, we carried out chemical and biological investigations of *L. abyssinica.* This present study focused on isolating and deciphering the structure of two steroids: sitost-5-en-3*β*-ol formiate (**1**), 5*α* and 6*β*-dihydroxysitosterol (**2**) and one triterpene: maslinic acid (**3**) along with the evaluation of their antibacterial and antifungal properties. This work also aimed at evaluating and finding any interactive relations between maslinic acid and sitost-5-en-3*β*-ol formiate isolated from *L. abyssinica* in the presence of either amoxicillin or fluconazole on MDR and reference strains as model organisms.

## 2. Materials and Methods

### 2.1. Spectroscopic and Chromatographic Analyses

Infrared (IR) spectra of isolated compounds were recorded with a Shimadzu FT-IR-8400S spectrophotometer (Shimadzu, France). Nuclear magnetic resonance (NMR) for ^1^H and ^13^C were recorded at 500 MHz and 125 MHz, respectively, with a Bruker Avance DRX-500 spectrometer (Bruker, Wissembourg, France) constituting a BBFO + 5 mm probe. NMR spectra measurement of ^1^H at 600 MHz and ^13^C at 150 MHz were performed with a tetramethylsilane (TMS) internal standard-built Bruker Avance III-600 spectrometer (Bruker, Wissembourg, France) using CD_3_OD as cryoplatform. Column chromatographic analysis was achieved through Merck silica gel with a pore size of 60 Å &70–230 mesh (VWR, France). Sephadex LH-20 chromatographic medium (VWR, France) was used for gel permeation chromatography. Thin layer chromatography was applied on precoated silica gel GF254. The plates were revealed by spraying with 10% of H_2_SO_4_ and heated at 100°C in an oven. Alternatively, using detection with ultraviolet visualization with the lamp at 254 and 365 nm was also fine.

### 2.2. Plant Material

*Ludwigia abyssinica* whole plant was obtained in Dschang, a university city in the West Region of Cameroon during the month of September 2016. Plant validity was confirmed with reference N° 40753/HCN in the National Herbarium in Yaounde.

### 2.3. Plant Component Obtention and Isolation

Five kilograms of the ambient-dried plant were crushed into powder and plant compounds were extracted with methanol (MeOH) for 72 hours. The extract was evaporated at low pressure and a 215 g dark crude extract was obtained. 210 g of this crude extract was dissolved in 400 ml of distilled water. Ethyl acetate (EtOAc) and *n*-butanol (*n*-BuOH) were used sequentially for further extraction of the dissolved extract. This yielded dried and concentrated ethyl acetate (80 g) and *n*-butanol (15 g) extracts. 75 g of EtOAc extract was purified over a silica gel column and hexane elution prepared with increasing EtOAc concentrations at 10 magnitude difference from 10 to 80%. Thin-layer chromatography was used to combine them in 9 subfractions: A, B, C, D, E, F, G, H, and I. Fraction B (3.58 g) was subjected over a silica gel column eluted and the hexane-EtOAc (90 : 10) purification yielded 14 g of compound **1.** The purification of fraction F (4 g) over a silica gel column eluted with a hexane-EtOAc mixture (60 : 40) gave 10 mg of compound **2**. The elution of fraction E (8 g) over a silica gel column chromatography with a hexane-EtOAc mixture (70 : 30) yielded 30 mg of compound **3**.

### 2.4. Antimicrobial Activity Test for Plant Components

#### 2.4.1. Test Organisms

Selected organisms for testing purified substances were three bacteria (*Staphylococcus aureus* ATCC 25923, *Escherichia coli* S2, and *Shigella flexneri* SDINT) and three yeasts (*Candida albicans* ATCC 10231, *Candida tropicalis* PK233, and *Cryptococcus neoformans* H99). All these microbes came from our laboratory bank of preserved microbes. These microbes were conserved at 4°C in nutrient agar (Conda, Madrid, Spain) for the bacteria and in Sabouraud dextrose agar (SDA, Conda, Spain) for yeasts before testing for antimicrobial properties.

#### 2.4.2. Investigation of Inhibitory and Microbicidal Concentrations

Antimicrobial activities of plant extracts and isolated compounds were assayed by analyzing their inhibitory, bactericidal, and fungicidal effects via minimum inhibitory concentrations (MICs), minimum bactericidal concentrations (MBCs), and minimum fungicidal concentrations (MFCs). The broth microdilution technique [18] with few modifications was used to establish MICs of the test samples. Obtained extracts/compounds were prepared in dimethylsulfoxide (DMSO, Fisher chemicals) to yield a solution stock. The latter was serially diluted two times in Sabouraud dextrose broth (SDB)/Mueller–Hinton broth (MHB) to attain concentration ranges of 512 to 0.50 *µ*g/mL and 4096 to 32 *µ*g/mL for extracts and isolated compounds, respectively. 10 *µ*L of bacteria (1 × 10^6^ CFU/mL) and 1 × 10^5^ spores/mL of yeast cells were prepared with each concentration in 90 *µ*L MHB or SDB culture medium to a final volume of 100 *µ*L. This yielded a final concentration span for pure compounds of 256 to 0.25 *µ*g/mL and 2048 to 16 *µ*g/mL for acquired extracts. Incubation ensued on a shaker are as follows: 24 h, 37°C for bacteria, 48 h, 30°C for *Candida* spp, and 72 h, 30°C for *Cryptococcus neoformans.* To determine MIC, 50 *μ*L of violet-colored *p*-iodonitrotetrazolium solution (0.2 mg/mL) was pipetted into wells and incubated for 30 minutes at 35°C. Viable microbes produced a pink coloration from the initial yellow one. The smallest sample concentration values (i.e. MIC) that altered this color change was considered to have offered maximum inhibition of bacteria or yeast growth. To establish minimum microbicidal concentration (MMC), 10 *µ*L of solution from the aliquots of growth-negative wells was added on MHA and SDA and incubation for each organism done respectively as indicated earlier. The least concentration for which no growth was observed after subculturing was considered as MBCs or MFCs [19]. The antibiotic positive control for the bacteria was amoxicillin and fluconazole for yeasts, and these two products were from Sigma-Aldrich in the city of Steinheim in Germany. The negative control used was 20 *µ*L of DMSO in the respective broth. Experiments were repeated thrice for each.

#### 2.4.3. Interactions between Antibiotics and the EtOAc Extract, Compounds **1** or **3**

The antimicrobial properties of an association of antibiotics (amoxicillin and fluconazole) with test samples (the EtOAc extract, compounds **1** and **3**), which showed the largest biological activity, were investigated using the checkerboard method as illustrated earlier [20]. The microbe seeding broth medium was made ready as directed earlier above. A plate of 96 wells was seeded with test microbes and stepwise dilutions were done with a pair of antimicrobial substances: antibiotic and the test sample. Each of the wells contained a mixture of the test sample with antibiotic at a defined concentration. Incubation of 96-well plates was done at 37°C for 24 h. MICs were obtained as indicated earlier above. All analyses were repeated three times. Interactive effects between antimicrobial substances were uncovered using the fractional inhibitory concentration (FIC) indices calculated as follows:

(1) $$\begin{array}{c} FIC=\frac{MIC\;of\;antibiotic\;tested\;in\;combination}{MIC\;of\;antibiotic\;tested\;alone}+\frac{MIC\;of\;extract\;or\;compound\;tested\;in\;combination}{MIC\;of\;extract\;or\;compound\;tested\;alone}\text{.} \end{array}$$

For FIC interpretation, a synergistic effect was indicated by a FIC value less than or equal to 0.5 (FIC ≤ 0.5). An addictive effect was identified when the FIC was less than or equal to 1 and more than 0.5 (0.5 < FIC ≤ 1). An indifferent effect was known when the FIC was less than or equal to 2 and more than 1 (1 < FIC ≤ 2). An antagonistic effect was declared when the FIC was above 2 (FIC > 2.0).

## 3. Results

### 3.1. Chemical Analysis

**Figure 1:**
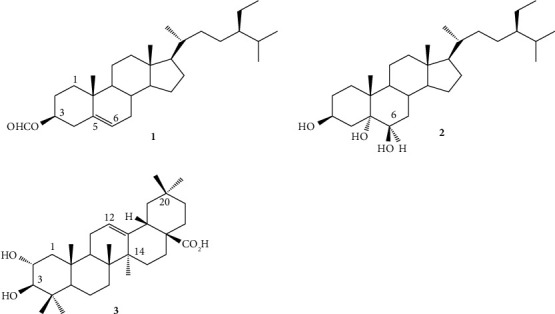
Structures of the isolated compounds from the whole plant of *Ludwigia abyssinica*.

In agreement with antimicrobial activities of the MeOH and EtOAc extracts, the EtOAc extract was further fractionated and purified. Three compounds were isolated and their structures (Figure 1) have been determined on the basis of spectroscopic data (^1^H and ^13^C NMR, ^1^H-^1^H COSY, HSQC, HMBC, ROESY, and NOESY) and by comparison to those published in the literature. Hence, the isolates were identified as sitost-5-en-3*β*-ol formiate (**1**) [21]; 5*α*,6*β*-dihydroxysitosterol (**2**), [22] and maslinic acid (**3**) [23] (Figure 1).

**Figure 2:**
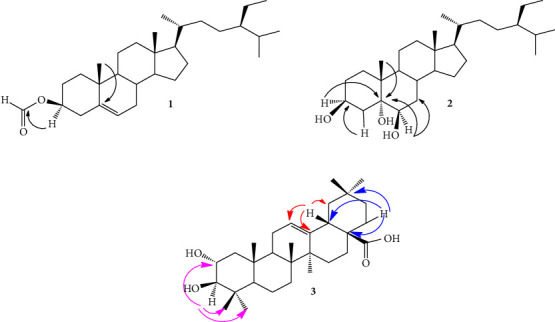
HMBC correlations of compounds **1**–**3**.

Sitost-5-en-3*β*-ol formiate (**1**): Colorless needles; C_30_H_50_O_2_; ^13^C NMR (100 MHz, CDCl_3_) *δ*_C_ 36.9 (C-1), 27.7 (C-2), 73.9 (C-3), 38.0 (C-4), 139.2 (C-5), 122.9 (C-6), 31.9 (C-7), 31.8 (C-8), 50.0 (C-9), 36.5 (C-10), 21.1 (C-11), 39.9 (C-12), 42.3 (C-13), 56.6 (C-14), 26.0 (C-15), 28.2 (C-16), 56.0 (C-17), 11.8 (C-18), 19.2 (C-19), 36.1 (C-20), 18.6 (C-21), 31.0 (C-22), 26.0 (C-23), 46.0 (C-24), 29.1 (C-25), 11.8 (C-26), 18.7 (C-27), 23.0 (C-28), 12.0 (C-29), 160.2 (OCHO, C-3). Its HMBC spectrum allowed us to confirm the sitostane skleton through the correlations observed between methyl groups and neighbouring carbons. This spectrum also confirmed the location of the formiate group at C-3 through the correlation observed between the proton H-3 at *δ*_*H*_ 4.76 ppm and the carbonyl group at *δ*_*C*_ 160.6 ppm (OCHO) (Figure 2).

5*α*,6*β*-dihydroxysitosterol (**2**): Colorless needles; C_29_H_50_O_3_; ^13^C NMR (100 MHz CDCl_3_): 34.2 (C-1), 30.2 (C-2), 75.4 (C-3), 40.4 (C-4), 75.8 (C-5), 67.2 (C-6), 32.2 (C-7), 30.8 (C-8), 45.4 (C-9), 38.0 (C-10), 21.0 (C-11), 36.0 (C-12), 42.7 (C-13), 56.1 (C-14), 24.6 (C-15), 28.4 (C-16), 55.8 (C-17), 11.8 (C-18), 16.5 (C-19), 30.1 (C-20), 19.3 (C-21), 33.8 (C-22), 24.0 (C-23), 45.7 (C-24), 29.1 (C-25), 18.9 (C-26), 19.6 (C-27), 22.9 (C-28), 12.0 (C-29). The correlations observed on its HMBC spectrum between the H-3 (3.42 ppm), H-19 (1.10 ppm), and the carbon C-5 (75.8 ppm) on the one hand and between H-6 (3.97 ppm) and carbons C-4 (40.4 ppm) and C-6 (67.2 ppm) on the other allowed us to locate the hydroxyl groups at C-3, C-5, and C-6.

Maslinic acid (**3**): White crystals; C_30_H_48_O_4_; ^13^C NMR (100 MHz CD_3_Cl_3_): 47.0 (C-1), 68.0 (C-2), 82.8 (C-3), 38.6 (C-4), 55.3 (C-5), 18.0 (C-6), 32.0 (C-7), 39.0 (C-8), 47.5 (C-9), 38.0 (C-10), 22.6 (C-11), 122.2 (C-12), 143.9 (C-13), 41.5 (C-14), 27.2 (C-15), 22.5 (C-16), 41.5 (C-17), 45.9 (C-18), 46.6 (C-19), 29.8 (C-20), 33.0 (C-21), 32.4 (C-22), 28.0 (C-23), 15.6 (C-24), 16.3 (C-25), 16.2 (C-26), 25.1 (C-27), 180.4, (C-28), 32.2 (C-29), 22.2 (C-30). The main correlations observed on its HMBC spectrum were in agreement with this structure and are shown in Figure 2.

### 3.2. Antimicrobial Activities

The antimicrobial activities of MeOH and EtOAc extracts and compounds isolated from the whole of *L. abyssinica* have been assessed against bacteria (*Shigella flexneri, Escherichia coli*, and *Staphylococcus aureus*) and yeasts (*Cryptococcus neoformans*, *Candida tropicalis*, and *Candida albicans*) (Table 1) selected based on their relevance as human pathogens. The extracts and isolates showed different MICs depending on the test microorganism (Table 1). The MICs of the MeOH and EtOAc extracts against bacteria and yeasts varied from 64 to 512 *µ*g/mL, demonstrating that the *L. abyssinica* can be a potent antimicrobial medicinal plant. Moreover, the antimicrobial activity of the EtOAc extract was greater than that of the MeOH extract. Compound **1** was the most active compound followed in decreasing order by compounds **2** and **3**. The antifungal and antibacterial properties of all the tested substances were lesser than those of fluconazole and amoxicillin used as reference drugs. The lowest MIC (16 *µ*g/mL) and MMC (32 *µ*g/mL) values were recorded with compound **1** against *S. aureus.* The largest MIC (512 *µ*g/mL) and MMC (1024 *µ*g/mL) values were noted against *C. albicans* with the MeOH extract. In general, the bacterial species were more sensitive to the extracts/isolates in comparison to the fungal species.

### 3.3. Association between Antibiotics and the EtOAc Extract, Compounds **1** or **3**

In order to determine the interaction effect between antibiotics and plant components (the plant extract and isolated compounds), FICIs were evaluated. The results (Table 2) showed an additive response in the case of compound **3** in combination with amoxicillin against multiresistant *E. coli* and *S. flexneri*. However, synergism was also recorded when this compound was used in conjunction with amoxicillin or fluconazole against *S. aureus*, *Candida albicans*, *C. tropicalis*, and *Cryptococcus neoformans*. Interestingly, the EtOAc extract and compound **1** displayed synergistic effects with antibiotics against all the tested pathogenic bacteria and yeasts. Among plant components-antibiotic combinations, the most effective were the EtOAc extract and amoxicillin (FICI = 0.093), compound **1** and amoxicillin (FICI = 0.156) against clinical strain *S. aureus* and the EtOAc extract and fluconazole, compound **1** and fluconazole against clinical strain *C. neoformans* (FICI = 0.093). Overall, the combinations between plant components and antibiotics showed 100% reduction in MICs of antibiotics when antibiotics were alone used.

## 4. Discussion

The present study shows antibacterial and antifungal activities of the MeOH extract from the whole plant *L. abyssinica*. These results corroborate those of the previous studies which reported the antifungal and antibacterial activities of the extracts from the *L. abyssinica* leaves [8, 17] and from different plant parts of *Ludwigia erecta* [24]. These findings also demonstrate that the antimicrobial activities of the EtOAc extract are greater than those of the MeOH extract. This difference in activities can be explained by the fact that the two solvents have different polarities and, therefore, their extracted metabolites would be different [25]. The antimicrobial properties of the isolates were generally higher than those of their extracts. These results are in agreement to those of Nzogong et al. [26] who showed an increase of the antimicrobial activity with the fractionation of extracts from the medicinal plants. Our results also demonstrate that the MICs of the plant components are four times lesser than the MBCs on the corresponding microorganisms, highlighting the microbicidal effects of most samples tested [25]. Plant extracts are consistently classified as antimicrobials on the basis of susceptibility tests that produce MIC values [27]. Hence, the MeOH extract of *L. abyssinica* was highly (MIC < 100 *μ*g/mL)/significantly (100 ≤ MIC ≤ 512 *μ*g/mL) active against the test bacterial species and significantly active against all the yeasts. The EtOAc extract was highly active against the test bacterial species and significantly active against yeasts. Interestingly, this study is the first to report on the isolation and structural elucidation of two steroids: sitost-5-en-3*β*-ol formiate (**1**) and 5*α*,6*β*-dihydroxysitosterol (**2**) and one triterpene: maslinic acid (**3**) with their antibacterial and antifungal properties. With the exception of compound **2** which demonstrated a moderate activity against *C. neoformans*, a low antifungal activity was noted with all compounds against *C. albicans, C. neoformans*, and *C. tropicalis* at the concentration range between 100 and 1000 *µ*g/mL. Based on the antimicrobial cutoff points defined in the literature [27], the antimicrobial activity of compounds **1** and **2** could be considered as moderate against bacteria and yeasts. Compound **3** exhibited a moderate activity against *E. coli, S. aureus S. flexneri*, and *C. neoformans* and a low activity against *C. tropicalis* and *C. albicans.* The presence of hydroxyl (-OH) and formyl (-OCHO) groups in positions 6 and 1 of compounds **1** and **2**, respectively, may explain the differences in the activity observed between these two compounds. The strains of *E. coli* S2 (1) and *Shigella flexneri* [28, 29] employed in this study are MDR clinical strains and they are resistant to commonly used antibiotics such as *co*-trimoxazole, tetracycline, streptomycin, ampicillin, furazolidone, and nalidixic acid. Most of the plant components displayed moderated antibacterial activities against these MDR strains, indicating that their administration may represent an alternative treatment against MDR *S. fexneri* and *E. coli*.

*Shigella* species, which are the causative organism of shigellosis, were formally sensitive to cotrimoxazole, chloramphenicol, ampicillin, and nalidixic acid but have presently become resistant to cephalosporins, azithromycin, and fluoroquinolones [30]. Most of the reported cases of shigellosis have been due to resistant strains of *Shigella* species. Emergent *E. coli* presenting different MDR phenotypes to three or more different antimicrobial agents have been reported and are responsible for serious health problems [31–33]. Hence, it is crucial to analyze multidrug-resistant*Shigella*/*Escherichia* and find new treatment modalities. In this work, we assessed the possibility of using *L. abyssinica* extract, maslinic acid (pentacyclic triterpenoid), and sitost-5-en-3*β*-ol formiate (steroid) as natural adjuvants for antibiotics against bacteria and fungi. FICI results depict an additive/synergistic relationship between maslinic acid and antibiotics. A synergistic effect was also observed between the plant extract/sitost-5-en-3*β*-ol formiate and antibiotics. The sum of the results shows that the activity of both plant extract/isolated compounds and antibiotics increased when they are combined with each other. These results are in conformity with the earlier findings where myrcene, *R*-limonene, *β*-elemene, sabinene, and *S*-limonene were found to be synergistic with first-line tuberculostatic antibiotics against isolated *Mycobacterium tuberculosis* [34]. Studies have reported the synergistic activity of numerous combinations of triterpenoids (rotundic acid, imberbic acid, ulsolic acid, betulinic acid, oleanolic acid, cycloastragenol, beta-boswellic acid, and ursolic acid) and antibiotics against multiresistant *Staphylococcus aureus* strains [6, 7]. To the best of our knowledge, this is a pioneer report on the plant extract/maslinic acid/sitost-5-en-3*β*-ol formiate and antibiotic associations against clinical strains.

The tested triterpene and steroids are relatively weaker than antibiotics. However, when assessed in association with antibiotics, they show relevant a synergistic effect and thus can help in prolonging the viability of these antibiotics against bacterial and fungal infections. In addition, reduction in the MIC value of amoxicillin and fluconazole with the plant extract, compounds **1** and **3** indicates their potential use against MDR *S. flexneri* and *E. coli*. The activity of antibiotics was further enhanced against bacteria and yeasts when these antibiotics were used in combination with the plant extract, compounds **1** or **3**. Test microorganisms were sensitive to amoxicillin and fluconazole when these antibiotics were mixed with plant components. Similarly, the amoxicillin activity was further increased against multiresistant *E. coli* and *S. flexneri* strains when combined with the plant components. Through MIC determinations, it was observed that both the plant extract/isolated compounds and antibiotics supplemented each other's effects. Amoxicillin is a beta-lactam antibiotic, which acts by binding to penicillin-binding proteins that inhibit the transpeptidation process, leading to the activation of autolytic enzymes in the bacterial cell wall [35], while fluconazole acts by inhibiting the conversion of lanosterol to ergosterol through the binding to fungal cytochrome P-450 leading to the disruption of fungal membranes [36]. The triterpenoids can theoretically act in a way that makes fungi/bacteria more sensitive to these antibiotics by destroying biofilms [37] or they can use some other pathways to exert the antimicrobial effect.

## 5. Conclusion

Overall, the results of this investigation demonstrate the antibacterial and antifungal activities of the extracts and compounds isolated from *L. abyssinica*. The EtOAc extract, compounds **1** and **3,** act in synergy with amoxicillin/fluconazole and thus can help in prolonging the viability of these antibiotics against bacterial and fungal infections, particularly those caused by multidrug resistant *E. coli* and *S. flexneri*.

## Figures and Tables

**Table 1:** Antimicrobial activity (MIC and MMC in *µ*g/mL) of *Ludwigia abyssinica* components and reference antibiotics.

Extracts/compounds	Inhibition parameters	*E. coli*	*S. flexneri*	*S. aureus*	*C. tropicalis*	*C. albicans*	*C. neoformans*
MeOH extract	MIC	128	128	64	256	512	128
	MMC	128	128	64	256	1024	256
	MMC/MIC	1	1	1	1	2	2

EtOAc extract	MIC	64	64	64	128	128	128
	MMC	128	64	64	256	256	128
	MMC/MIC	2	1	1	2	2	1

**1**	MIC	32	32	16	64	64	32
	MMC	64	64	32	>256	>256	64
	MMC/MIC	2	2	2	—	—	2

**2**	MIC	32	64	128	64	64	32
	MMC	64	128	>256	>256	>256	>256
	MMC/MIC	2	2	—	—	—	—

**3**	MIC	64	64	32	64	128	64
	MMC	128	128	64	>256	>256	>256
	MMC/MIC	2	2	2	—	—	—

Ref^*∗*^	MIC	16	16	1	0.5	1	2
	MMC	16	16	1	0.5	1	2
	MMC/MIC	1	1	1	1	1	1

—: not determined; MIC: minimum inhibitory concentration; MMC minimum microbicidal concentration; ^*∗*^: fluconazole and amoxycillin for yeasts and bacteria, respectively.

**Table 2:** Interactions of *L. abyssinica* components and antibiotics against bacteria and yeasts.

Microorganisms	*EtOAc extract*				*Compound * ***1***				*Compound * ***3***			
	FICA	FICEx	FICI	Interpretation	FICA	FIC2	FICI	Interpretation	FICA	FIC5	FICI	Interpretation
*E. coli*	0.125	0.125	0.375	Synergistic	0.25	0.125	0.375	Synergistic	0.50	0.25	0.75	Additive
*S. flexneri*	0.125	0.062	0.187	Synergistic	0.25	0.125	0.375	Synergistic	0.50	0.125	0.625	Additive
*S. aureus*	0.062	0.031	0.093	Synergistic	0.125	0.031	0.156	Synergistic	0.25	0.125	0.375	Synergistic
*C. tropicalis*	0.25	0.125	0.375	Synergistic	0.25	0.125	0.375	Synergistic	0.25	0.25	0.50	Synergistic
*C. albicans*	0.125	0.125	0.25	Synergistic	0.125	0.062	0.187	Synergistic	0.25	0.125	0.375	Synergistic
*C. neoformans*	0.062	0.031	0.093	Synergistic	0.062	0.031	0.093	Synergistic	0.25	0.125	0.375	Synergistic

FICA: MIC of antibiotic tested in combination/MIC of antibiotic tested alone; FICEx: MIC of extract tested in combination/MIC of extract tested alone; FIC2: MIC of compound **1** tested in combination with antibiotic/MIC of compound **1** tested alone; FIC4: MIC of compound **3** tested in combination with antibiotic/MIC of compound **3** tested alone; FIC: MIC of antibiotic tested in combination/MIC of antibiotic tested alone + MIC of extract/compound tested in combination/MIC of extract/compound tested alone; antibiotics: ciprofloxacin for bacteria and fluconazole for yeasts.

## Data Availability

The datasets used and analyzed to support the findings of this study are available from the corresponding authors upon request.

## Abbreviations

^13^C-NMR: Carbon thirteen nuclear magnetic resonance
^1^H-NMR: Proton nuclear magnetic resonance
2D NMR: Two-dimension nuclear magnetic resonance
ATCC: American Type Culture Collection
CC: Column chromatography
COSY: Correlation spectroscopy
DMSO: Dimethylsulfoxide
EtOAc: Ethyl acetate
HMBC: Heteronuclear multiple bond connectivities
HNC: Herbier National du Cameroun
HR-EI-MS: High resolution electron impact mass spectrometry
HR-TOFESIMS: High-resolution time of flight electrospray ionization mass spectrometry
HSQC: The heteronuclear single quantum coherence
IR: Infrared
MBC: Minimum bactericidal concentration
MDR: Multidrug resistant
MeOH: Methanol
MHA: Mueller–Hinton agar
MHB: Mueller–Hinton broth
MIC: Minimum inhibitory concentration
MMC: Minimum microbicidal Concentration
NA: Nutrient agar
n-BuOH: n-Butanol
NMR: Nuclear magnetic resonance
Rf: Retention factor
TLC: Thin-layer chromatography
TMS: Tetramethylsilane
TOF-ESIMS: Time of flight electrospray ionization mass spectrometry
UV: Ultraviolet.
